# Supplementary material for: Observational evidence of increased afternoon rainfall downwind of irrigated areas
Source: Nat Commun. 2025 Apr 10;16:3415. doi: 10.1038/s41467-025-58729-y (PMC11986030; doi:10.1038/s41467-025-58729-y)
Supplement: Supplementary file 1 — Supplementary Information [file 41467_2025_58729_MOESM1_ESM.pdf]

Supplementary Information: Observational Evidence of Increased Afternoon Rainfall Downwind of Irrigated Areas

P. Greve[1], A. U. Schmitt[1], D. G. Miralles[2], S. McDermid[3,4], K. L. Findell[5], A. García-García[6,7], J. Peng[6,7]

[1]Climate Service Center Germany (GERICS), Helmholtz-Zentrum Hereon, Hamburg, Germany
[2]Hydro-Climate Extremes Lab (H–CEL), Ghent University, Ghent, Belgium
[3]Department of Environmental Studies, New York University, New York, NY, USA
[4]NASA Goddard Institute for Space Studies, New York, NY, USA
[5]Geophysical Fluid Dynamics Laboratory (GFDL), National Oceanic and Atmospheric Administration (NOAA), Princeton, USA
[6]Department of Remote Sensing, Helmholtz Centre for Environmental Research–UFZ, Leipzig, Germany
[7]Remote Sensing Centre for Earth System Research, Leipzig University, Leipzig, Germany

March 24, 2025

Supplementary Figures

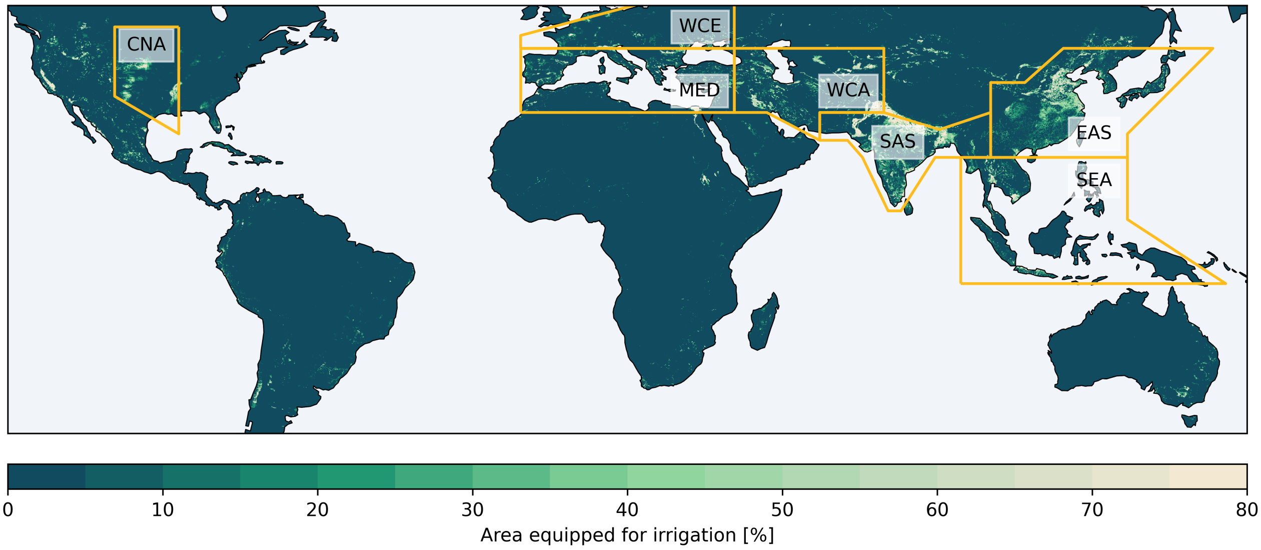

Figure S1: Global map showing the percentage of area equipped for irrigation (AEI) within each grid cell. IPCC regions featuring the highest number of events are highlighted (see also Fig. 2).

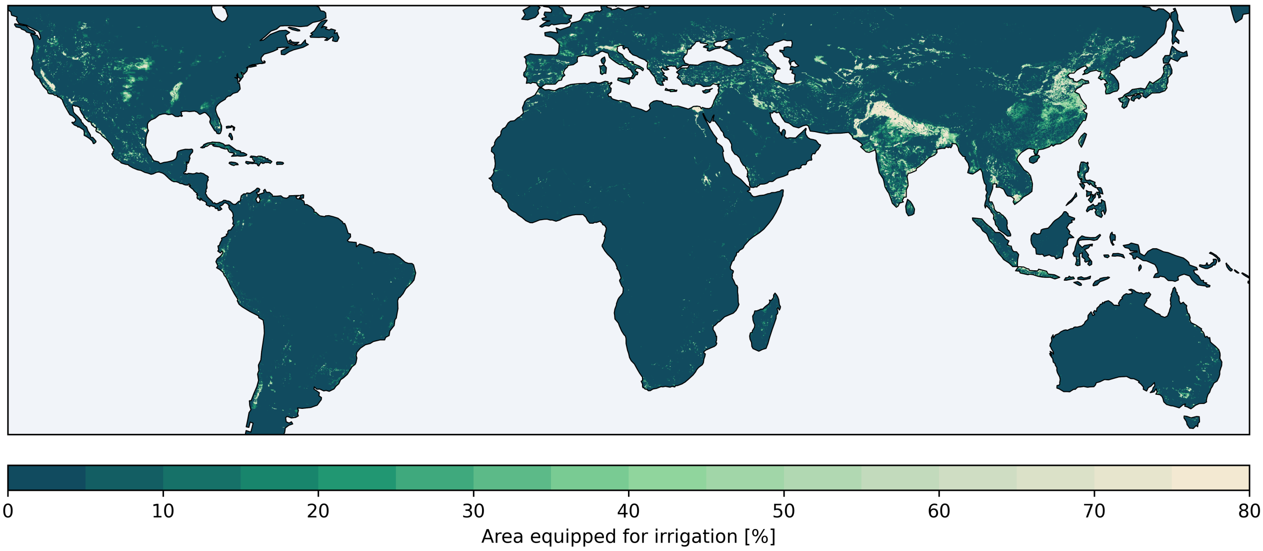

Figure S2: Global map showing the percentage of area equipped for irrigation (AEI) within each grid cell.

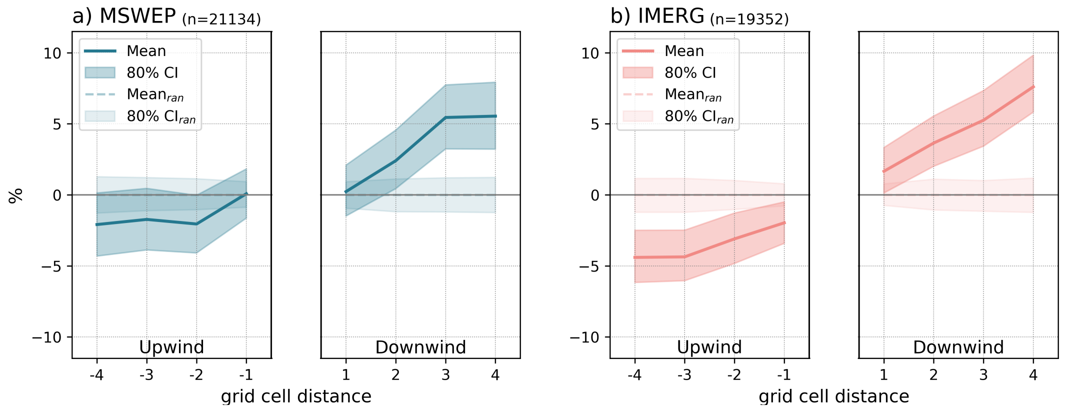

Figure S3: Average likelihood of peak afternoon rain along the wind direction (same as in Fig. 3c,d) normalized by randomized wind directions. This means that the average of the randomized wind directions is subtracted from the average likelihood and 80% confidence interval (shaded area). The dashed line (zero due to normalization) and the pale shading indicate the average and the 80% CI for the randomized wind directions (CI_{ran}).

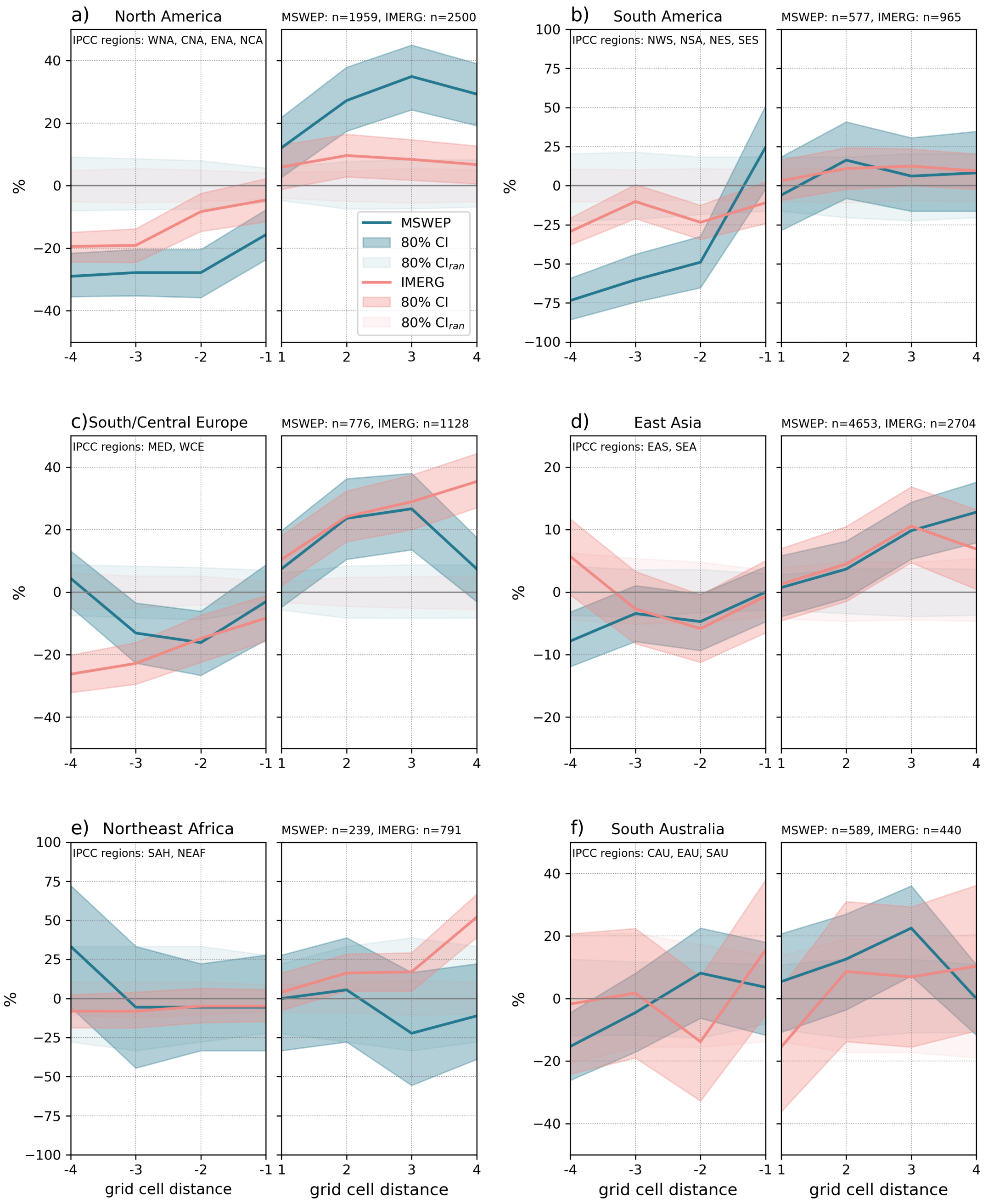

Figure S4: Regional differences in peak rainfall likelihood: Average likelihood of peak afternoon rain along the wind direction normalized by randomized wind directions (same as in Fig. S3) for rain events detected in (a) North America, (b) South America, (c) South/Central Europe, (d) East Asia, (e) Northeast Africa and (f) South Australia. The shaded area represents the 80% confidence interval (CI) based on a bootstrapping approach. The dashed line (zero due to normalization) and the pale shading indicate the average and the 80% CI for the randomized wind directions (CI_{ran}). Please note that the sample sizes, detected relative differences, and their associated uncertainties vary between the regions (note the different y-axes).

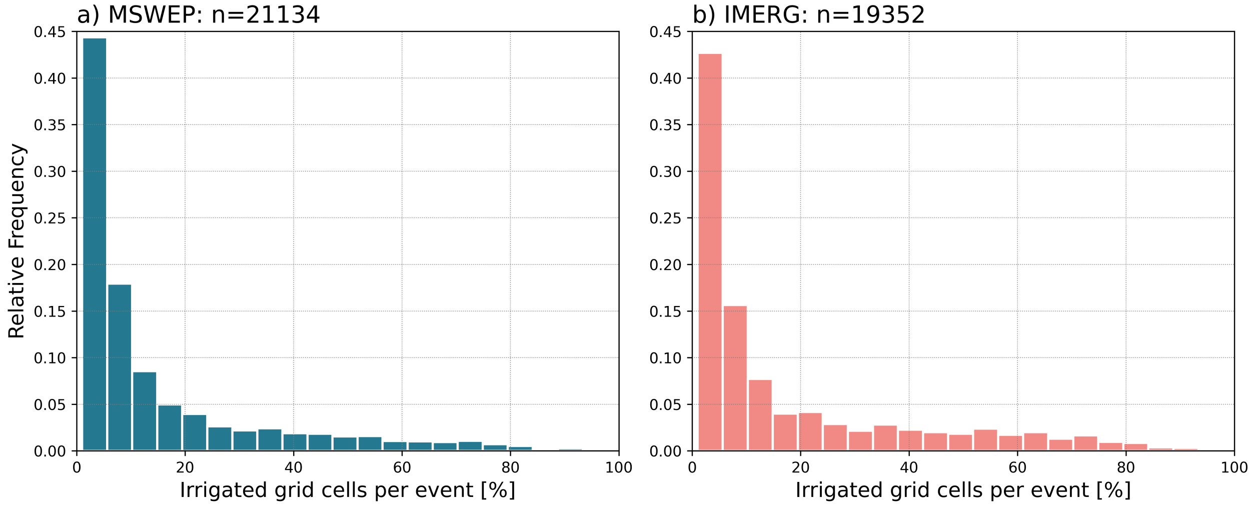

Figure S5: Relative frequency of irrigated grid cells considering all rain events for (a) MSWEP, and (b) IMERG. Ca. 75% for MSWEP and ca. 70% for IMERG of all rain events feature less than 20% irrigated grid cells within a $50km$ radius surrounding the peak location

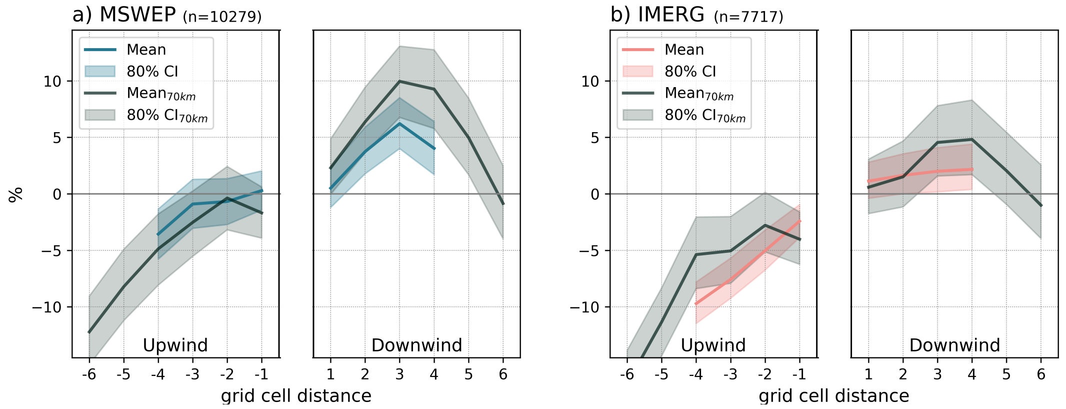

Figure S6: Average likelihood of peak afternoon rain along the wind direction (same as in Fig. 3c,d) and the average likelihood estimated by considering a 70km radius surrounding peak afternoon rain locations (70km, dark gray colors). The shaded area represents the 80% confidence interval (CI) based on a bootstrapping approach.

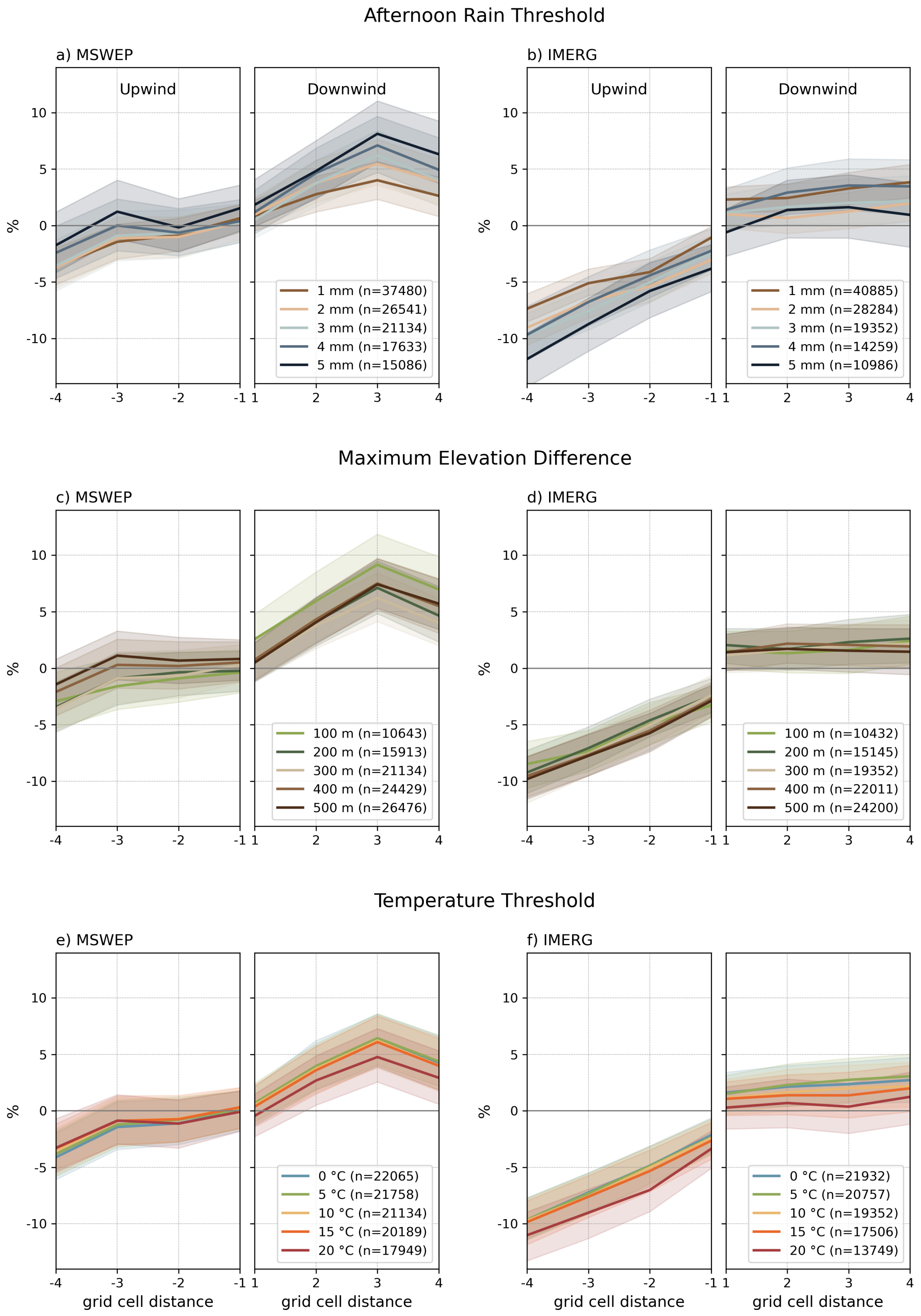

Figure S7: Average likelihood of irrigated grid cells along the wind direction (same as in Fig. 3) for MSWEP and IMERG rain data under varying afternoon rain thresholds (a,b), maximum elevation differences (c,d), and temperature thresholds (e,f). The shaded areas represent the 80% confidence interval (CI) based on a bootstrapping approach.

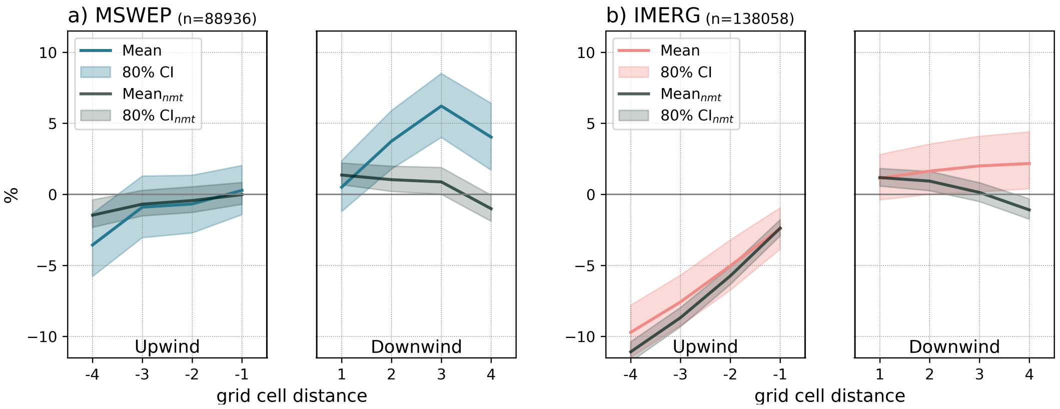

Figure S8: Average likelihood of peak afternoon rain along the wind direction (same as in Fig. 3c,d) and the average likelihood estimated from all detected afternoon rain events without necessarily considering a dry morning (nmt = no morning threshold, dark gray colors). The shaded area represents the 80% confidence interval (CI) based on a bootstrapping approach.

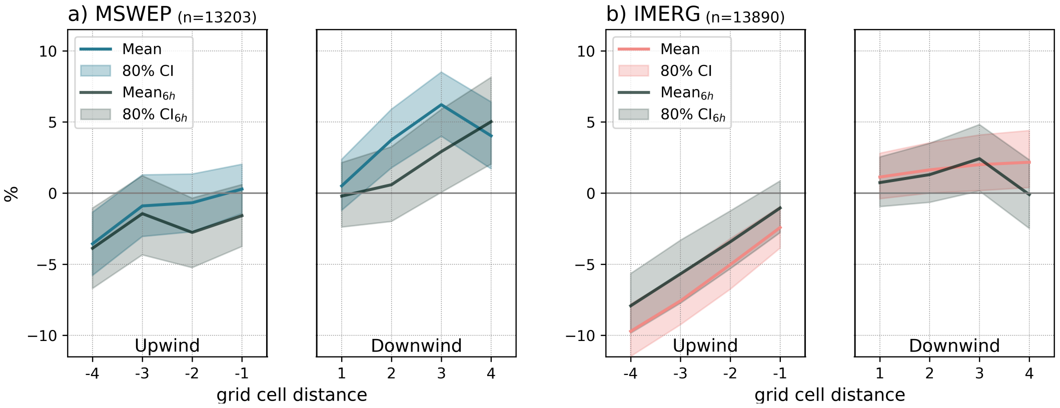

Figure S9: Average likelihood of peak afternoon rain along the wind direction (same as in Fig. 3c,d) and the average likelihood estimated from all detected afternoon rain events considering a six-hourly afternoon from 12h to 18h ($6h$ = six-hourly afternoon, dark gray colors). The shaded area represents the 80% confidence interval (CI) based on a bootstrapping approach.

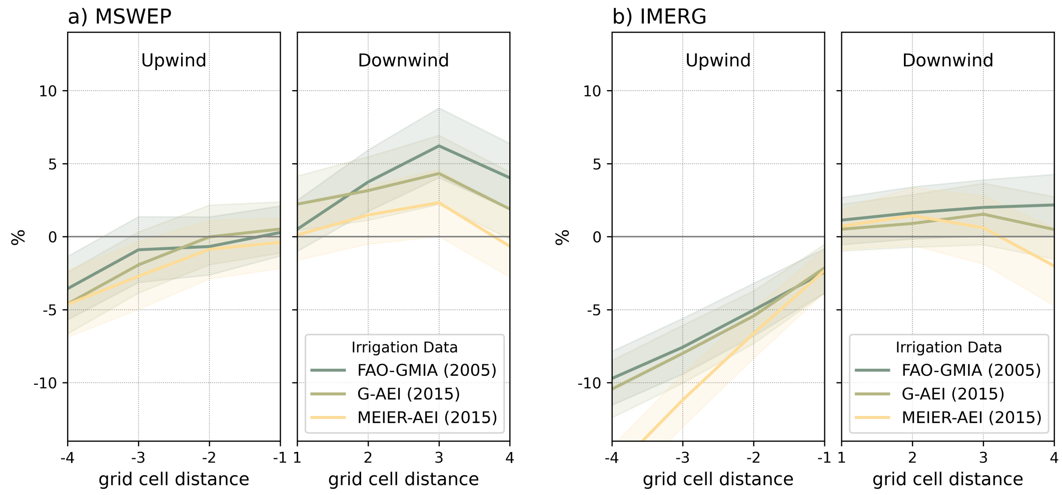

Figure S10: Average likelihood of irrigated grid cells along the wind direction (same as in Fig. 3 for FAO-GMIA) for MSWEP and IMERG rain data considering two additional irrigation datasets: (i) Global Area Equipped for Irrigation Dataset for the year 2015 (G-AEI)[48] and (ii) Global Irrigated Areas for the year 2015 (MEIER-AEI)[49]. The shaded areas represent the 80% confidence interval (CI) based on a bootstrapping approach.

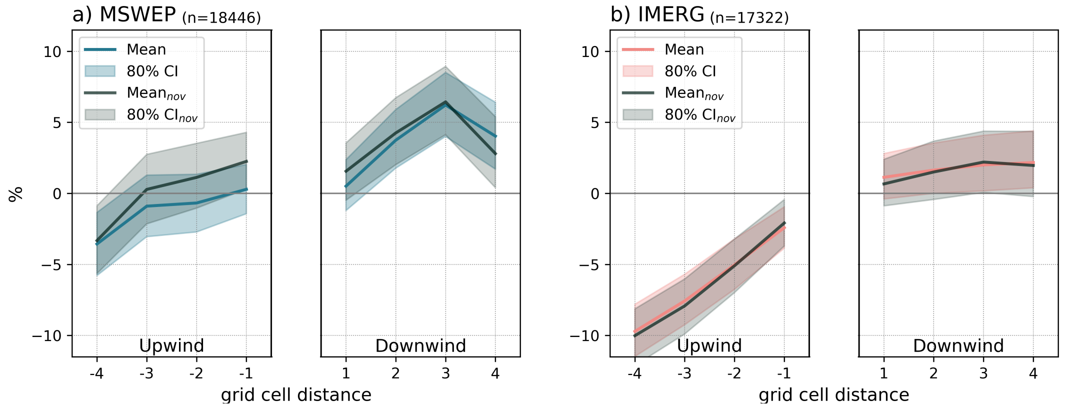

Figure S11: Average likelihood of peak afternoon rain along the wind direction (same as in Fig. 3c,d) and the average likelihood estimated by excluding overlapping events (nov = no overlapping, dark gray colors). The shaded area represents the 80% confidence interval (CI) based on a bootstrapping approach.

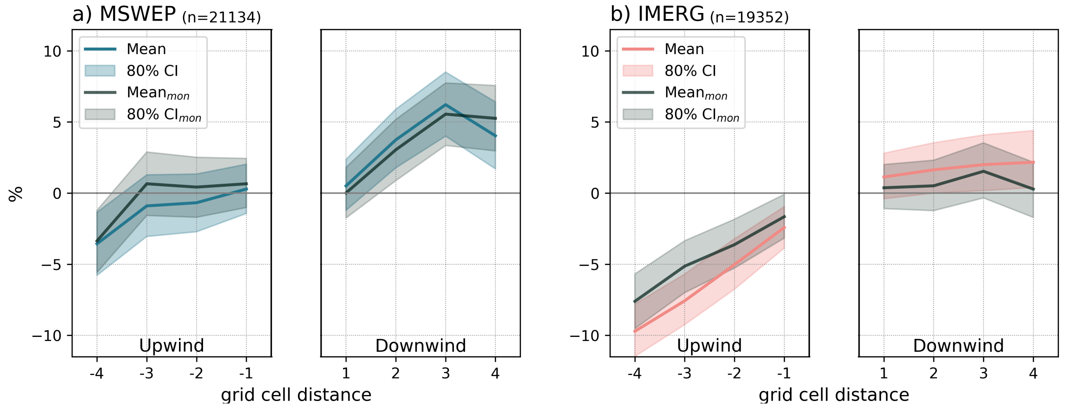

Figure S12: Average likelihood of peak afternoon rain along the wind direction (same as in Fig. 3c,d) and considering wind directions based on actual mean monthly wind data from ERA5 from 2001 to 2020 (dark gray colors). The shaded area represents the 80% confidence interval (CI) based on a bootstrapping approach.

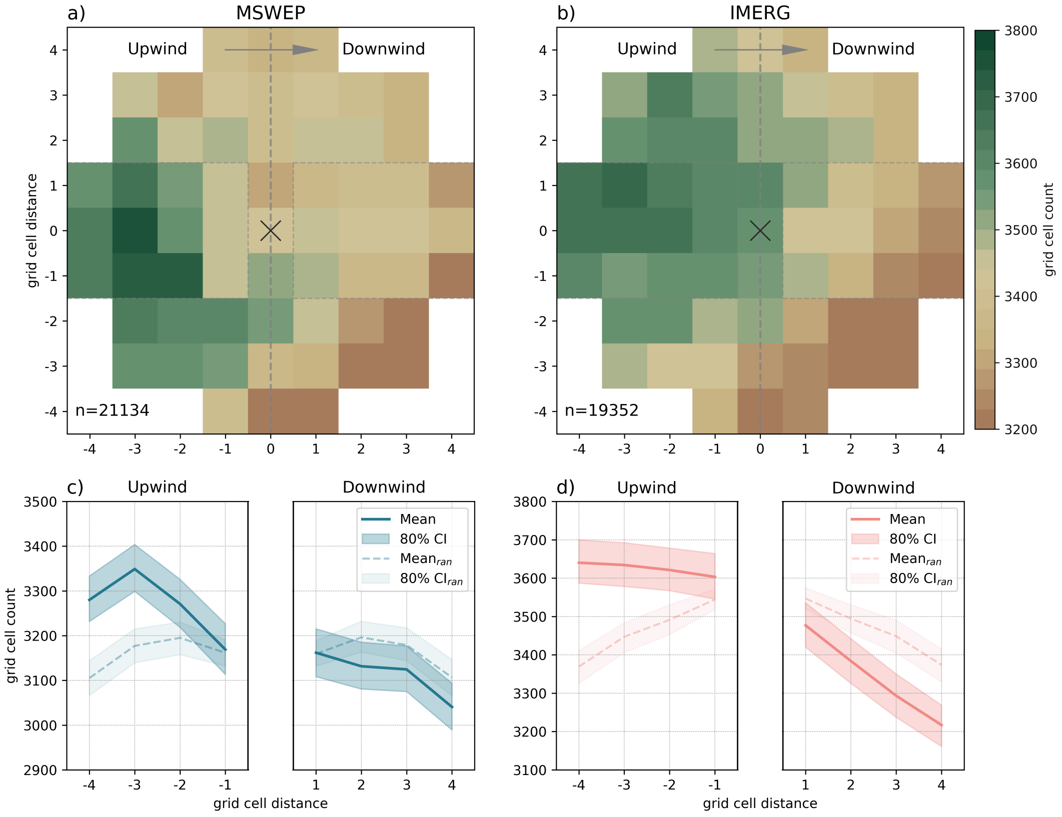

Figure S13: Sum of irrigated grid cells across all rain events relative to the location of peak afternoon rain. (a, b) Raster maps showing the sum of irrigated grid cells relative to the location of peak afternoon rain centred at (0,0) for MSWEP and IMERG rain data. Wind direction is normalized such that upwind (downwind) areas are on the left (right). In comparison to Fig. 3, within this reference system centred around the peak afternoon rain location, grid locations to the left of the centre define areas upwind of peak afternoon rainfall, i.e., the peak location is located downwind of these areas. Similarly, grid locations to the right of the centre define areas downwind of peak afternoon rainfall, i.e., the peak rain location is located upwind of these areas. (c, d) Average sum of irrigated grid cells along the wind direction (+/-1 grid cells, within the dashed horizontal lines in a, b). The shaded area represents the 80% confidence interval (CI) based on a bootstrapping approach. The dashed line and the pale shading indicate the average and the 80% CI for the randomized wind directions (CI_{ran}).

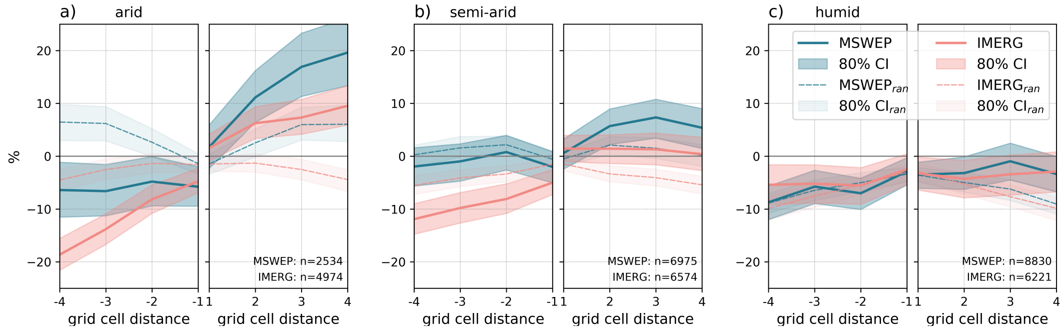

Figure S14: Average likelihood of peak afternoon rain along the wind direction (same as in Fig. 3) for rain events detected in (a) arid ($AI < 0.2$), (b) semi-arid ($0.2 < AI < 0.5$), and (c) humid ($AI > 0.5$) regions. The shaded area represents the 80% confidence interval (CI) based on a bootstrapping approach. The dashed lines indicate the $80\% CI$ for the randomized wind directions (CI_{ran}).

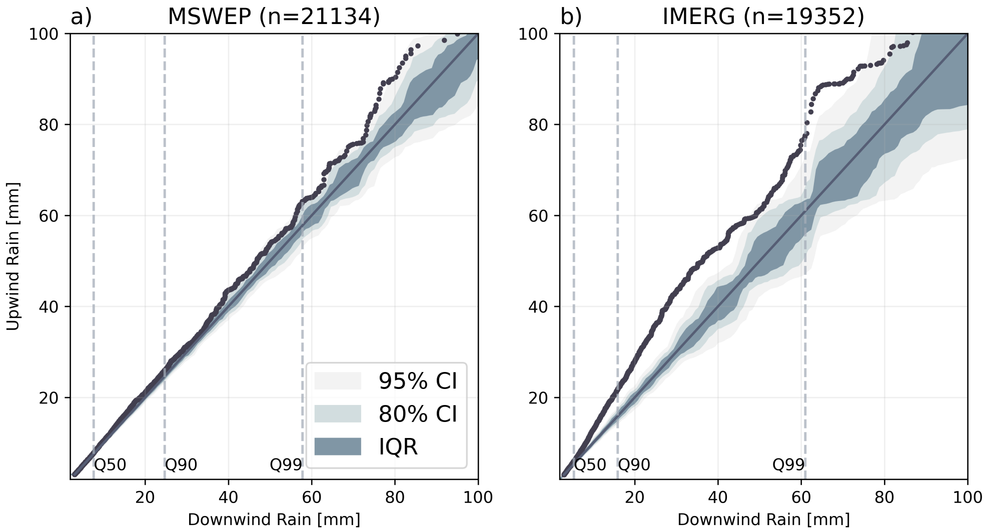

Figure S15: Quantile–Quantile plots of peak rain intensity for rain events downwind (x-axis) and upwind of irrigated grid cells (y-axis) considering (a) MSWEP, and (b) IMERG rain data.Here we only consider those events as upwind events that are outside the entire downwind half. Individual gray dots represent rainfall events. The shading represents the uncertainty range obtained by choosing 1000 equally sized random samples. The vertical dashed lines illustrate the 50th, 90th and 99th quantile.

Supplementary Tables

Percentile	1	5	10	25	50	75	90	95	99
MSWEP	3.06	3.25	3.56	4.63	7.63	14.0	24.5	33.25	57.64
IMERG	3.03	3.15	3.31	3.87	5.34	8.72	15.8	24.3	61.02

Table S1: Percentiles (in mm) of sample distributions of afternoon rainfall events detected using MSWEP and IMERG.
